# Fat replacement by pecan nut and oregano oil and their impact on the physicochemical properties and consumer acceptability of frankfurters

**DOI:** 10.5713/ab.20.0622

**Published:** 2020-12-01

**Authors:** Daniela Orozco, Alma Delia Alarcon–Rojo, Celia Chavez-Mendoza, Lorena Luna, Luis Manuel Carrillo-Lopez, Oswaldo Ronquillo

**Affiliations:** 1 Faculty of Animal Science and Ecology, Autonomous University of Chihuahua, Chihuahua, Chih., 31453, Mexico; 2 Centre for Food Research and Development, A.C. (CIAD), Delicias, Chih., 33089, Mexico; 3 CONACYT, Faculty of Animal Science and Ecology, Autonomous University of Chihuahua, Chihuahua, Chih., 31453, Mexico; 4 National Institute for Forest, Agriculture and Livestock Research (INIFAP). La Campana experimental site, Aldama, Chih., 32910, Mexico

**Keywords:** Consumer Acceptability, Fat Replacement, Frankfurters, Oregano Oil, Pecan Nut, Physicochemical Properties

## Abstract

**Objective:**

A study was conducted to determine the physicochemical quality and consumer acceptability of beef frankfurter-type sausages formulated with pecan nut paste and Mexican oregano oil (MO) of two varieties, *Poliomintha longiflora* Gray (Mexican oregano *Poliomintha*, MOP) or *Lippia berlandieri* (Mexican oregano *Lippia*, MOL).

**Methods:**

Frankfurters were processed under six treatment conditions: control (10.67% pork fat), MOP (control + 0.01% MOP), MOL (control + 0.01% MOL), MOP-N (control + 0.01% MOP + 6% pecan nut paste), MOL-N (control + 0.01% MOL + 6% pecan nut paste), and C-N (control + 6% pecan nut paste). The physicochemical properties and the consumer acceptability were determined.

**Results:**

The addition of MO and nut did not influence the water holding capacity, drip loss, and luminosity of frankfurters, but those ingredients increased pH and shear force (p<0.05) and decreased redness (p<0.05) of frankfurters. Frankfurters were generally well accepted by consumers. However, general acceptability of sausages decreased with the addition of MO. Control sausages showed the highest acceptability.

**Conclusion:**

The MO and pecan nut paste do not affect drastically the quality of frankfurters. These results provide evidence that partial replacement of pork fat by pecan nut in frankfurters maintain a consistent physicochemical quality and its commercialization looks promising given consumers' acceptance.

## INTRODUCTION

The trend of consuming low fat, healthier and functional foods has stimulated the meat industry to develop novel meat product formulations that replace the fat of animal origin with vegetable oil and additionally antioxidant ingredients have been proposed to offer a healthier alternative for consumers [1,2]. The use of vegetable oil to substitute the animal fat totally or partially could provide the meat products with natural antioxidants and modulate some specific physiological functions [3] promoting the consumer health.

Among the healthy alternatives for fat substitution in meat emulsions, walnut oil can be an option as it contains considerable amounts of antioxidants and unsaturated and polyunsaturated fatty acids (PUFAs). It is known that tree nuts are an outstanding source of no-saturated fatty acids and other nutrients including vitamin E, vitamins and minerals, such as folate, potassium, magnesium, as well as bioactive compounds related to human health [4]. A representative family of nut trees is the Juglandaceae genera of tree nuts including walnuts and pecan (*Carya illinoinensis* [Wangenh.] C. Koch), their nuts are very popular all over the world.

Essential oils, including oregano oil are natural additives used in the processing and preservation of diverse foods including meat products. Oregano is widely used as flavouring agent of Mediterranean, Asian, and Latin America cuisines. However, some authors reported that essential oils have the disadvantage of residual aromas and flavours.

Emulsified meat products usually have up to 30% animal fat, thus the addition of antioxidants during manufacture is a common practice to avoid rancidity and low sensory quality. A recommended option is to add essential oils as a natural ingredient to prevent lipid oxidation. In a previous work we have reported that the combination of pecan nut with Mexican oregano essential oil (*Lippia berlandieri* [*L. berlandieri*]) could be a good strategy for increasing the ratio of unsaturated fatty acids to saturated fatty acids without compromising on frankfurter quality [5]. When new ingredients are added, the technological and sensory characteristics of the new-formulated meat products should be studied to ensure consumer acceptance of the products. Recently, some research articles showed the use of oleogels as replacers of animal fat to formulate novel processed meat products. Frankfurters have been manufactured with sunflower oil [6] and linseed [7] as fat replacers with variable results. To develop products for health-conscious customers some researchers have proposed the use of oleogels with omega-3 and PUFA’s [8] as healthy fat substitutes. Whereas other authors have used enriched vegetable oleogels as replacers of animal fat in the bologna-type formulations [9], meat patties [10], and pâtés [11]. However, products designed to better meet technological properties and consumer expectations have yet to be created.

Animal fat is needed to provide textural characteristics of frankfurters and stabilization of the emulsion. Besides, animal fat provides flavor, juiciness, and a desired mouth feel of frankfurters. Previous studies in our laboratory [5] showed that the use of 100 ppm (0.01%) oregano oil and 6% pecan nut paste in frankfurter formulation produce good quality frankfurters. However, tests of consumer sensory acceptability of frankfurters made with this formulation have not been carried out.

The replacement of pork fat with pecan nut paste and oregano essential oil in the frankfurter formulation could enhance the nutritive value of the product by increasing the antioxidant capacity, and the content of unsaturated and polyunsaturated fatty acids. In this way, the innovative product will satisfy the needs of the consumer by offering a healthier and functional innovative product. Therefore, the present study was aimed to evaluate the effect of the partial replacement of pork fat with pecan nut paste and oregano essential oil on the physicochemical properties and consumer acceptability of frankfurters.

## MATERIALS AND METHODS

### Treatments

Six formulations of frankfurter sausages were prepared with or without Mexican oregano essential oil (MO) of two varieties (*Poliomintha longiflora* [*P. longiflora*] Gray, MOP; and *L. berlandieri*, MOL) and pecan nut (*Carya illinoinensis* K. Koch) paste. The formulations were: i) control (traditional formulation, 10.67% pork fat); MOP (control + 0.01% MOP); ii) MOL (control + 0.01% MOL); iii) MOP-N (control + 0.01% MOP + 6% pecan nut paste), iv) MOL-N (control + 0.01% MOL + 6% pecan nut paste); and v) C-N (control + 6% pecan nut paste). When 6% pecan nut paste was added, only 4.67% pork fat was used in formulation (Table 1).

### Nut paste preparation

Pecan nuts used in this study were seeds of *C. illinoinensis* (K. Koch) Western variety. The nuts were obtained from a local producer in central-southern of Chihuahua state, Mexico. For preparing pecan nut paste, nuts were shelled and then ground using a colloidal mill until a creamy paste was formed. The mean particle size of 40 microns. The obtained milled pecan nut paste was used as partial fat replacer in the formulations of frankfurters.

### Oregano essential oil

Oregano (*P. longiflora* Gray and *L. berlandieri* Schauer) was obtained from a local producer in central-southern of Chihuahua state, Mexico. The leaves and stems of the plant were used for extraction of essential oil. The oil was obtained by steam distillation for 3 h. The essential oil was dried using anhydrous sodium sulfate and stored in an amber bottle at 4°C until further use.

### Frankfurter preparation

For preparation of the frankfurter, frozen (−12°C) beef (neck and forearm) and pork fat were obtained from a local market. Meat (71.32%) at 2°C was cut into approximately 1-cm^3^ cubes and placed in a Hobart cutter (Model 84145; Hobart Corporation, Troy, OH, USA) for 3 min. Next, salt (1.42%), nitrites (0.014%), sodium ascorbate (for the control; 0.03%), and 2/3 ice (9.6%) were slowly added. Tripolyphosphates (0.14%), condiments (0.35%), L-glutamic acid monosodium salt (0.2%), sugar (0.64%), and 1/3 ice (4.8%) were incorporated and mixed for 2 min (temperature of the emulsion 3°C±1°C). Fat (10.67%) was added at 2°C, and the mixture was ground for 2 min. In all treatments, the total amount of fat was constant (10.67%); when 6% pecan nut paste was added, only 4.67% pork fat was used. The essential oil (0.01%) and pecan nut paste (0% or 6.0%) were added according to each treatment formulation. The meat emulsion was stuffed into cellulose casing (3 cm in diameter) using a meat stuffer (Model M-22 R1; TorRey, Mexico). The sausages were manually tied with cotton wool every 15 cm in length, vacuum packed, and subjected to thermal heating in a water bath (Precision Scientific Thelco Model 85; Waltham, MA, USA) until reaching an internal temperature of 70°C±2°C. Next, the sausages were cooled in an ice-water bath for 20 min and drained for 10 min. Frankfurters were packed and stored at 4°C until analysis. The experimental unit was six sausages per treatment, and determinations were performed in triplicate.

### Physicochemical analyses

Frankfurter samples were examined to determine pH, colour coordinates (L*, a*, and b*), water holding capacity (WHC), drip loss (DL), and shear force (SF). All physicochemical variables were measured at the same day and they were determined in triplicate.

#### Colour and pH evaluation

The CIE L*a*b* color coordinates were measured with a Chromameter (Konica Minolta, CR 400, Chiyoda, Tokio, Japan) standardized with a white plate (L* = 95.87, a* = −0.49, b* = 2.39 and an aperture of 8 mm). Prior to the measurement the sample was exposed to oxygen for 10 min. The colour coordinate values represented L* (brightness/darkness), a* (redness/greenness), and b* (yellowness/ blueness) according to AMSA [12]. pH was measured with a digital portable meat pH meter (Model 1001; Sentron Technologies, Roden, The Netherlands) with the electrode inserted into the center of the meat and pH readings were recorded at three locations along the sausage sample.

#### Water holding capacity and drip loss

Measurement of WHC was performed following the compression technique of Honikel and Hamm [13] with the modifications. A sample of 0.3 g (±0.05 g) was placed between two filter paper (Whatman 41) and a weight pressure of 5 kg was applied for 20 min. The WHC was determined by calculating the weight difference before and after being pressed and expressed as a percentage. Measurement of DL was made in a sample of 3 g of the cooked sausage and expressed as percentage loss weight of the initial weight.

#### Evaluation of shear force

The SF was determined using the methodology of the AMSA [12]. Samples were plastic-bag packed and cooked by immersion in a water bath (Isotemp 215; Fisher Scientific, Pittsburgh, PA, USA) for the time needed for the temperature at the center of the sample to reach 72°C; then they were stored at 4°C for 24 h. After this period, eight cylinders of 10 mm diameter were cut using a food drill. Cores were cut with a TA-XT2i (Stable Micro Systems, Surrey, UK) adapted with a Warner–Bratzler V-shaped blade attached to a 100 N load cell. The crosshead speed used was 200 mm/min. Average values for each sample were determined, and the SF values were reported as Newtons.

### Acceptability test

The acceptability of the frankfurters was evaluated by an untrained consumer panel. The consumers (panellists) who participated in the acceptability test, were 65 undergraduate or postgraduate students of the Faculty of Animal Science and Ecology, with an age range was 18 to 23. The recruitment process was conducted via classroom invitation. Consumers were recruited based on the following criteria: i) like of frankfurters, ii) consuming processed meat products at least twice a week, and iii) interest in participating in the study. The analysis was performed in the Sensory Analysis Laboratory (20°C) equipped with 12 cabins and white light (fluorescent) was used to observe the appearance of the samples. The acceptability tests were performed in one 30 min session.

Samples of sausages of approximately 3 cm length, previously identified with a random number of three digits, were placed in glass dishes, and offer to the panellists in a random order. Panellists were asked to take spring water and salt-free bread to cleanse their palates between tastings. Panelists assessed a total of six frankfurter samples, from the 6 experimental treatments and were free to re-taste as needed to allow them to make comparisons among samples and confirm their assessment. Consumers were instructed to taste and evaluate the frankfurters based on the odour, flavour, texture, colour, and overall acceptability. Participants evaluated samples on a 7-point hedonic scale (1, dislike extremely; 2, dislike moderately; 3, dislike slightly; 4, neither like nor dislike; 5, like slightly; 6, like moderately; 7, like extremely) [14] for scoring and global satisfaction degree of the frankfurter samples.

The present study was submitted to and approved by the Institutional Bioethics Committee of the Faculty of Animal Science and Ecology of the Autonomous University of Chihuahua, Mexico (Decision No. P/302/2017). The entire procedure adopted in the sensory test was explained in detail to the panellists taking part in the analyses, who signed a free and informed consent form.

### Statistical analysis

Data were analysed in a completely randomized design (SAS Institute Inc., Cary, NC, USA). Comparisons of means were made using Tukey tests, and an alpha level less than or equal to 0.05 was set to accept statistical differences. Correspondence analysis was applied to generate a biplot to visualize the relationship between treatments and their sensory attribute liking using XLSTAT-Pro (Addinsoft Inc., Long Island City, NY, USA) trial version.

## RESULTS AND DISCUSSION

### Physicochemical characteristics

The addition of pecan nut paste in meat emulsions could not only offer possible health benefits, but also provide important effects on sensory and technological quality. Considering that sausages are a multiple emulsion and according to the nature of the organic, oily, and aqueous phases that comprise it, significant differences are expected in their organoleptic and physicochemical characteristics. Hydrogen potential (pH), WHC, DL, color (L*, a*, b*, C*, and h*), and SF are indicators of the quality of fresh meat and meat products [15].

In previous work we showed that the partial replacement of fat with pecan nut paste added to oregano oil does not change the proximate composition of frankfurters [5]. The average values for moisture, protein, fat, and ash content of sausages of all the treatments are 69.39%, 14.89%, 8.17%, and 3.71%, respectively.

The results of the physicochemical analyses are presented in Table 2. The addition of pecan nut paste to frankfurters increased (p<0.05) the pH, potentially due to the pH of pecan nuts. Several authors have reported increases in pH of meat products due to the addition of peanut paste [16], while others did not find significant changes in the pH of frankfurters made with walnuts [17]. A basic parameter of meat quality assessment is pH, since it directly affects the stability and properties of proteins as well as quality attributes such as WHC, DL, and color of meat [15]. Also, the pH is responsible for the quality of the products, a decrease in pH causes denaturation of muscle proteins and a shrinkage of the polypeptide chain network that leads to a decrease in the ability of meat to retain water. On the other hand, an increase in the pH causes an increase in the WHC of the meat due to the changes in the electrical charges of the muscle proteins [15]. In the present study the pH of the frankfurtes increased suggesting an improvement in the quality of the product.

No differences (p>0.05) were found in the WHC or DL of the six treatments in this study (Table 2). Thus, replacing 6.0% fat in the original frankfurter formulation did not affect the water retention of the product. A recent study [18] shows that sausages containing walnut and olive oil had lower cooking losses and higher emulsion stability than control sausages. These authors prepared chicken sausage with the addition of PUFAs containing 2.5 g/100 g walnut in their formula. WHC is defined as the ability of meat or muscle proteins to firmly retain and/or mobilize meat's own or added water during the application of a known force. Some characteristics of meat that are linked to WHC are color, texture, tenderness, juiciness, and DL [15]. The increase in pH observed in the frankfurters did not cause a change in the WHC of the product. Probably that differences in pH value was not enough to cause significant changes in the muscle proteins of polypeptide network that retain water.

Colour is crucial in both, quality, and acceptability of meat products. The colour parameters of frankfurters are shown in Table 2. Analysis of colour parameters displayed that the luminosity (L*) was not significantly affected (p>0.05) by the addition of MO and pecan nut paste. Similarly, other authors reported no changes in L* of sausages with 3% oregano essential oil [19].

The addition of pecan nut paste decreased (p<0.05) the redness (a*) of the sausages, whereas the MO did not significantly affect this parameter. The dilution of the meat pigments responsible for colour by the outer water of the meat lattices contributes to lower the colour parameters due to a different concentration of myoglobin. This is the case of the frankfurters containing nut and/or MO which lack of the red colour of meat myoglobin. In addition, the pecan paste, and the MO are mixed with muscle proteins and water form an emulsion which in turn causes a light dispersion, that can also cause a change in colour. These findings were consistent with the results of Baek et al [20], who replaced animal fat with canola and flaxseed oils and observed a decrease in redness of chicken sausages. The results of the present study confirm that the replacement of animal fat with vegetable oils results in a decrease in the redness of the meat products.

The yellowness (b*) of frankfurters was different (p<0.05) between treatments. In general, sausages that contained both, MO, and pecan nut paste, had greater yellowness than the control sausages. This could be due to the presence of red tannins from the nuts [21]. The effect of vegetable ingredients on the colour of meat products has clearly been presented by Youssef and Barbut [22] who explained that the during the emulsion making, the distribution of oil into the protein matrix results in colour changes due to differences in particle area (bigger in fat than in oil particles). Furthermore, vegetable oil is homogenous and more easily dispersed in an emulsion than fat of animal origin.

Besides the effect of pecan nut paste, the impact of MO on frankfurter colour should also be considered. Thus, the increase in b* value of sausages containing MO could also be due to the brownish-yellow colour of the oregano oil as reported by Cao et al [23]. These authors prepared inulin/chitosan blend films containing oregano and thyme essential oils and found important changes in the film colour with a decrease in L* and increased in a* and b* values. Al-Hijazeen et al [24] and Al-Hijazeen et al [25] have reported that oregano essential oil improved the stability of the raw and cooked chicken meat colour with better colour values (L* and a*) than untreated meat. The increase in b* agrees with previous studies for meat products manufactured with linseed oil [7]. Da Costa et al [19] reported the effect of oregano and Rosemary essential oils on the quality of chicken sausages. They evaluated the colour of sausages both, externally and internally. The results showed a significant increase (p<0.05), during storage, for L*, a*, and b* values. This possibly occurred because of the presence of the essential oil that contributed to their antioxidant properties. Moreover, they reported no differences in colour between treatments with essential oil and control during storage of sausages. Similar results were reported in cooked ham prepared with pecan and peanut paste [26].

Frankfurters made with MOP (Oregano [*P. longiflora* Gray] essential oil) were tougher (p<0.05) than the control with lower SF values. However, no difference was observed in the rest of the treatments, which showed similar (p>0.05) SF values to the control and to the MOP treatment. In general, the SF tended to increase slightly when animal fat was replaced with MO and pecan nut paste (Table 1). It has been reported that some vegetable oils help to restore textural parameters that are modified when animal fat is reduced in comminuted meat products. That is the case of the study of Youssef and Barbut [22] who substituted beef fat with canola oil in comminuted meat products. They observed that reducing fat from 25% to 10% decreased hardness, but textural parameters of the products were restored by the addition of canola oil. Similar results were reported by Baek et al [20] who observed that the texture profile of sausages manufactured with canola oil showed the highest profile values for texture among the treatments studied. The results published are very variable and it may be due to the properties of the oil and the product formulation itself. In a recent work, pork fat in frankfurters was replaced by soybean oil oleogels structured with rice bran wax and no effects (p>0.05) were found on the texture profile of sausages [27]. In general, texture of frankfurters made with vegetable oil is softer than those products made with animal fat since oil emulsify better than solid fats commonly used in the manufacture of meat products, therefore, the end product could have better texture.

### Acceptability

Colour, texture, flavour, and aroma of frankfurters are crucial attributes in purchase decision and they are critical factors influencing consumer satisfaction. Consumers were presented with traditional frankfurters (control), frankfurters prepared with two species of MO, and frankfurters prepared with MO and pecan nut paste, and they then scored different characteristics of each product. The consumer acceptability of frankfurters is presented in Table 3. The partial substitution of pork fat with pecan nut paste and MO decreased the acceptance of colour, texture, and flavour of the frankfurters (p<0.05). Panellists scored the treated samples (with MO and/or N) lower than the control for colour, texture, and flavour, however, scores were not dramatically lower than the control (Table 3). Frankfurters containing pecan nut paste and MO did not change the aroma scores given by the panellists as no significant difference (p>0.05) was observed in that attribute. The concentration of oregano essential oil (0.01%) used in this study was not high enough to negatively affect the odour of frankfurters. While low (0.01%) concentration of oregano oil influenced the acceptability of frankfurter flavour, higher concentration would be needed to influence the acceptability of frankfurter odour.

**Figure 1:**
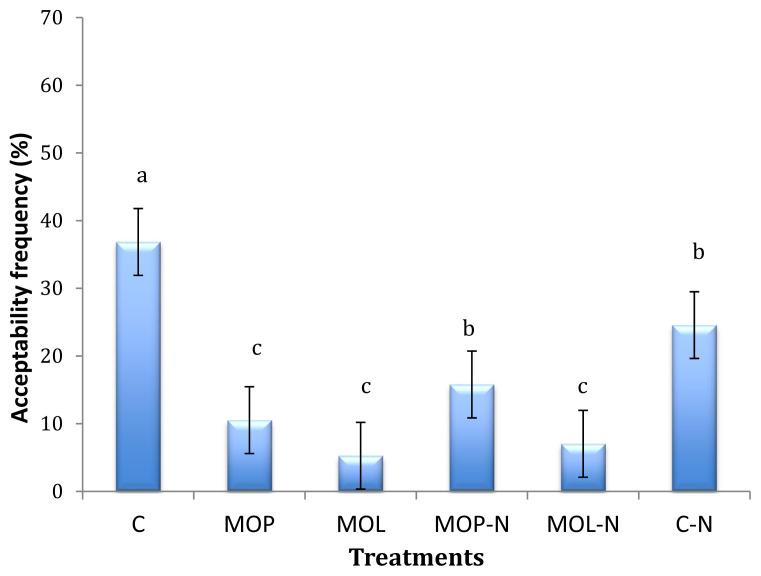
Realtive frequency of acceptability of Frankfurters made with two varieties of oregano (*Poliomintha longiflora* Gray and *Lippia berlanderi* Schauer) essential oil and pecan nut paste and stored at 0 and 7 days at 4°C. C, control; MOP, oregano (*Poliomintha longiflora* Gray) essential oil; MOL, oregano (*Lippia berlanderi*) essential oil; MOP-N, oregano (*Poliomintha longiflora* Gray) essential oil + 6% pecan nut paste; MOL-N, oregano (*Lippia berlanderi*) essential oil + 6% pecan nut paste; C-N, control + 6% pecan nut paste. ^a–c^ Means are different (p<0.05).

When MO was added to the frankfurters, the overall acceptability decreased (Figure 1). The control sausage showed the highest acceptability (37%), followed by the sausage containing 6% pecan nut paste without oregano oil (C-N) (25%), while the sausages prepared with MOP [oregano (*P. longiflora* Gray) essential oil] showed the lowest consumer acceptability (5%). However, when pecan nut paste was added together with MOL, the acceptability increased. This might indicate that oregano oil flavour and odour compounds negatively affect the acceptability of frankfurters. (Figure 1). The higher acceptability of the control frankfurter could be attributed to its conventional characteristics of a commercial sausage. It is important to take into account that the development of new meat products containing new ingredients should consider evaluating sensory traits and consumer acceptability.

It has been reported that when peanut paste, pecan nuts, and walnuts were added to ham, the acceptability decreased [26]. Also, the low acceptability could rely on the oregano oil compounds. The highest amount of volatiles components present in oregano are carvacrol and thymol [5]. However, Al-Hijazeen et al [25] observed that a 200 ppm oregano oil combined with 10 ppm of tannic acid presented high score on the overall acceptability of the cooked chicken meats.

Some researchers observed that sensory parameters could not be substantially improved when pork back fat was replaced with a linseed oleogel [7]. In another study, Yildiz-Turp and Serdaroglu [28] reported the effect of replacing beef fat with hazelnut oil in the production of emulsified meat and observed a reduction in quality due to lipid instability and a risk of oxidation of the product containing hazelnut oil. Also, the development of healthier lipid pâtés with beeswax oleogels as fat replacer showed no significant effects on sensory quality, whereas substitution by ethyl cellulose had an undesirable effect and the authors assumed that it was due to the substitution level [11]. It is, however, important to mention that some reports found no changes in the acceptability of fat-replaced meat products [6,29]. Da Silva et al [9] demonstrated that replacing of up to 50% of pork back fat by oleogel prepared with pork skin and sunflower oil did not affect the sensory quality of bologna-type sausages.

Furthermore, there are reports showing that burger acceptability increased with the addition of healthy fat replacers. Martins et al [8] showed that hamburgers enriched with omega-3 and PUFAs oleogels were high scored during sensory evaluation, but the panellists preferred the control samples. Contrarily to this study, Oh et al [10] found that the replacement of beef tallow with hydroxypropyl methylcellulose containing canola oil oleogels for meat patties showed a good overall consumer acceptability at the 50% replacement level, and finally, a recent study of low fat PUFAs-enriched pork burgers made with oleogels and curcumin as an antioxidant showed that Beeswax oleogel presented good overall sensory acceptability [11]. These reports provide evidence that research on fat replacement with oleogels enriched with PUFAs or other natural antioxidants requires further work regarding sensorial acceptability.

**Figure 2:**
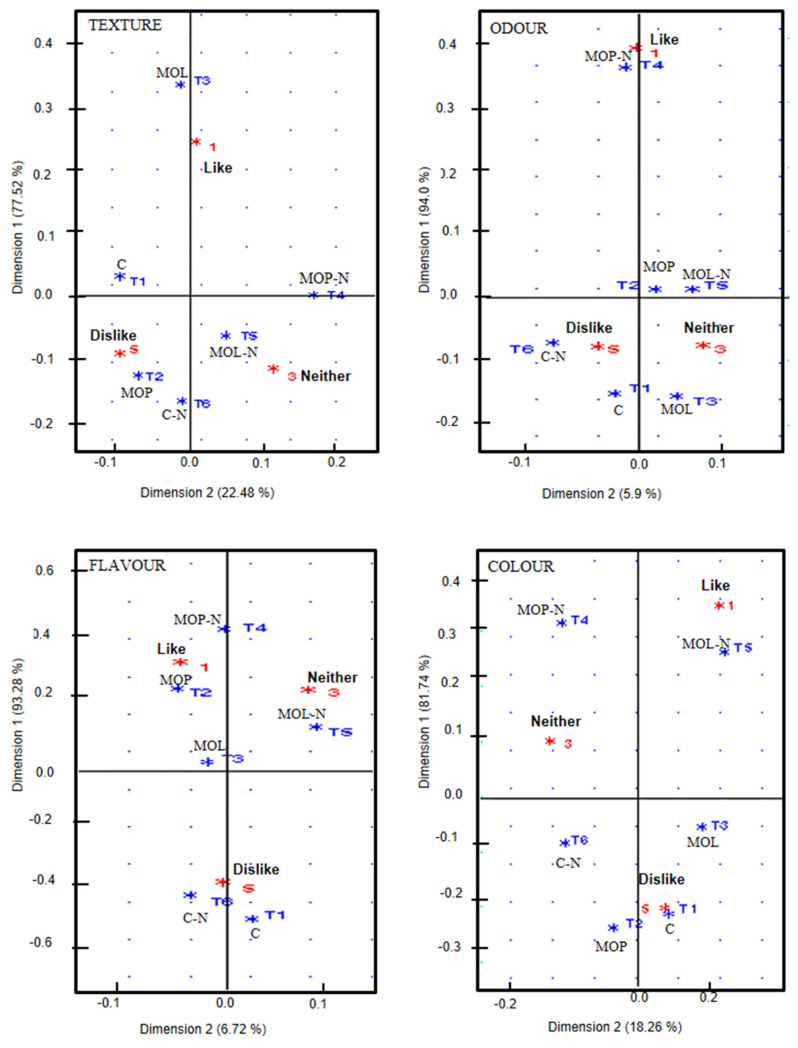
Correspondence analysis plot based on the treatments and the attribute acceptability of Frankfurters measured in the consumer test. C, control; MOP, oregano (*Poliomintha longiflora* Gray) essential oil; MOL, oregano (*Lippia berlanderi*) essential oil; MOP-N, oregano (*Poliomintha longiflora* Gray) essential oil + 6% pecan nut paste; MOL-N, oregano (*Lippia berlanderi*) essential oil + 6% pecan nut paste; C-N, control + 6% pecan nut paste.

Correspondence analysis is an exploratory data analysis tool that not only helps to identify the existence of relationship between variables but also shows how variables are related. The correspondence analysis plot based on the treatments and the attribute acceptability measured in the consumer test is shown in Figure 2. The two axes accounted for 100% of the inertia or total variance for texture (dimension 1, 77.52% and dimension 2, 22.48%), 99.9% of the total variance for odour (dimension 1, 94.0% and dimension 2, 5.9%), and 100% of the total variance for flavour (dimension 1, 93.28% and dimension 2, 6.72%) and colour (dimension 1, 81.74% and dimension 2, 18.26%). Practically, the total variance could be explained in the first dimension. Therefore, the association between MO levels and/or pecan nut paste and the sensory attributes in sausages is one-dimensional, indicating that it contributed to a greater extent to the discrimination of the treatments, grouping them according to the degree of acceptability by the consumer. Moreover, significant differences in the acceptability of the attributes existed between treatments.

Texture was directly correlated with MOL, which was the most acceptable treatment, but was negatively correlated with MOP and CN (control + 6% pecan nut paste), which were the less acceptable treatments. In contrast, odour and flavour were majorly explained by MOP-N (mexican oregano Poliomintha oil + pecan nut), whereas colour was majorly explained by MOL-N (mexican oregano Lippia oil + pecan nut), which had the highest acceptability. This result suggested that sausages manufactured with oregano oil, with or without pecan nut, contributed the most to the acceptability attribute. As it is known, fat plays an important role in the sensory characteristics of meat products, thus, any change in the quantity or type of fat in formulation may give place to changes in quality properties [30].

The results of the overall liking test showed that MO-containing frankfurters were less acceptable than control sausages which could be attributed to differences between individual attributes and the acceptability of the whole product. These findings also indicate that acceptability is a complex attribute, and that analysis of individual attributes does not necessarily correlate with overall liking. In general, the attributes of colour, texture, and odour were all well accepted, whereas acceptability of flavour was low.

Sensory quality depends on the interaction between the ingredients of the formulation for the development of texture and mouth feel of a food. In addition, the quality of technology also is the result of these interactions in the emulsion that can affect changes in its stability. A reduced stability of the emulsion would mean eventually a phase separation, which in practice, supposes the breakage of the emulsion and, therefore, a final product of low performance and quality, economic losses, and consumer rejection.

The fiber (6.7 g/100 g) and protein content (16.7 g/100 g) of walnut paste used in the formulation of Frankfurt sausages produces interactions between fiber binders and fat-protein during the emulsification process that allows low values of total expressible fluids and fat exudates. This means a high capacity to retain water and fatty exudates, indicates better emulsion stability and rheological properties [17].

## CONCLUSION

The inclusion of 0.01% of two varieties of MO (*P. longiflora* Gray and *L. berlandieri*) and 6% pecan nut paste in frankfurters resulted in minimal changes in physicochemical characteristics. However, the addition of both species of oregano essential oil resulted in lower consumer acceptability. Traditional frankfurter formulation (control) showed the highest consumer acceptability followed by the treatment containing 6% pecan nut paste without oregano oil. Therefore, pecan nut paste can be used in the formulation of healthier frankfurters without compromising on physicochemical quality of the final product and acceptable reduced-fat products can be produced when back fat is replaced with up to 6% pecan nut paste. Possibly, reducing the concentration of MO during product formulation will help to achieve a well-balanced product, satisfying consumers' acceptability requirements. These findings indicate that pecan nut can be used in the formulation of frankfurter to offer an acceptable product to health-conscious consumers.

## Figures and Tables

**Table 1:** Ingredients (g/100 g) used in the formulation of Frankfurter-type sausages

Ingredient (%)	Treatment^1)^					

	C	MOP	MOL	MOP-N	MOL-N	C-N
Meat	71.32	71.32	71.32	71.32	71.32	71.32
Ice	14.50	14.50	14.50	14.50	14.50	14.50
Fat	10.67	10.67	10.67	4.67	4.67	4.67
Salt (NaCl) (1.18)	1.420	1.420	1.420	1.420	1.420	1.420
Alginate	0.710	0.710	0.710	0.710	0.710	0.710
Sugar (0.18)	0.640	0.640	0.640	0.640	0.640	0.640
Condiment (0.52)	0.350	0.350	0.350	0.350	0.350	0.350
Glutamic acid	0.200	0.200	0.200	0.200	0.200	0.200
Tripolyphosphates^2)^	0.140	0.140	0.140	0.140	0.140	0.140
Nitrite	0.140	0.140	0.140	0.140	0.140	0.140
Ascorbate	0.030	0.030	0.030	0.030	0.030	0.030
Oregano essential oil	-	0.010	0.010	0.010	0.010	0
Nut paste	-	-	-	6.00	6.00	6.00

1) C, control; MOP, oregano (*Poliomintha longiflora* Gray) essential oil; MOL, oregano (*Lippia berlanderi*) essential oil; MOP-N, Oregano (*Poliomintha longiflora* Gray) essential oil + 6% pecan nut paste; MOL-N, oregano (*Lippia berlanderi*) essential oil + 6% pecan nut paste; C-N, control + 6% pecan nut paste.

2) Monosodium salt.

**Table 2:** Physicochemical characteristics of Frankfurters (means±standard deviation) made with two varieties of oregano (*Poliomintha longiflora* Gray and *Lippia berlanderi* Schauer) essential oil and pecan nut paste

Characteristic^2)^	Treatment^1)^					

	C	MOP	MOL	MOP-N	MOL-N	C-N
pH	5.96±0.11^b^	6.01±0.08^b^	5.99±0.13^b^	6.28±0.16^a^	6.30±0.12^a^	6.28±0.14^a^
WHC (%)	68.40±0.0^a^	64.60±8.77^a^	67.30±2.12^a^	59.60±0.68^a^	58.30±2.36^a^	59.60±3.5^a^
DL (%)	6.07±1.13^a^	8.54±0.96^a^	7.47±1.43^a^	8.99±0.54^a^	5.78±1.48^a^	7.88±1.22^a^
L*	46.00±1.14^a^	48.40±1.88^a^	46.00±1.15^a^	47.80±2.22^a^	47.40±0.54^a^	49.70±2.33^a^
a*	12.40±0.95^a^	12.30±0.86^a^	12.50±1.32^a^	9.39±0.96^b^	10.10±0.47^b^	9.51±0.89^b^
b*	7.82±0.11^b^	10.60±0.45^a^	9.96±0.51^a^	10.40±0.06^a^	10.70±0.42^a^	10.60±0.27^a^
SF (N)	5.98±0.3^b^	11.60±1.04^a^	7.31±0.9^b^	7.69±1.59^b^	8.47±2.79^b^	7.61±0.64^b^

1) C, control; MOP, oregano (*Poliomintha longiflora* Gray) essential oil; MOL, oregano (*Lippia berlanderi*) essential oil; MOP-N, oregano (*Poliomintha longiflora* Gray) essential oil + 6% pecan nut paste; MOL-N, oregano (*Lippia berlanderi*) essential oil + 6% pecan nut paste; C-N, control + 6% pecan nut paste.

2) WHC, water holding capacity; DL, drip loss; L*, luminosity; a*, redness; b*, yellowness; SF, Warner Bratzler shear force (N).

a,b Different letters in the same row mean significant difference (p<0.05).

**Table 3:** Sensory evaluation of acceptability of Frankfurter-type sausages (means±standard deviation) made with two varieties of oregano (*Poliomintha longiflora* Gray and *Lippia berlanderi* Schauer) essential oil and pecan nut paste

Treatments^1)^	Sensory attributes^2)^			

	Colour	Texture	Flavour	Odour
C	4.76±1.58^a^	4.72±1.34^a^	4.85±1.41^a^	4.89±1.22^a^
MOP	4.76±1.38^a^	4.29±1.35^b^	3.85±1.57^c^	4.65±1.25^a^
MOL	4.50±1.53^b^	3.77±1.65^b^	4.15±1.64^c^	4.72±1.32^a^
MOP-N	4.08±1.26^b^	4.00±1.36^b^	3.60±1.44^c^	4.38±1.54^a^
MOL-N	4.03±1.35^b^	4.18±1.42^b^	4.05±1.57^c^	4.57±1.45^a^
C-N	4.60±1.49^b^	4.29±1.44^b^	4.74±1.72^b^	4.80±1.45^a^

1) C, control; MOP, oregano (Poliomintha longiflora Gray) essential oil; MOL, oregano (Lippia berlanderi) essential oil; MOP-N, oregano (Poliomintha longiflora Gray) essential oil + 6% pecan nut paste; MOL-N, oregano (Lippia berlanderi) essential oil + 6% pecan nut paste; C-N, control + 6% pecan nut paste.

2) Acceptability was evaluated on a 7-point hedonic scale (1, dislike extremely; 2, dislike moderately; 3, dislike slightly; 4, neither like nor dislike; 5, like slightly; 6, like moderately; 7, like extremely).

a–c Different letters in the same column mean significant difference (p<0.05).
